# A Readmission Risk Model for Hospitalized Patients Receiving Dialysis: Evaluation of Predictive Performance

**DOI:** 10.1016/j.xkme.2022.100507

**Published:** 2022-06-24

**Authors:** David M. Gallagher, Congwen Zhao, Benjamin A. Goldstein

**Affiliations:** 1 Hospital Medicine Programs, Duke University School of Medicine, Durham, NC; 2 Department of Biostatistics and Bioinformatics, Duke University, Durham, NC; 3 Department of Biostatistics and Bioinformatics, Center for Predictive Medicine, Duke Clinical Research Institute, Duke University, Durham, NC

To the Editor:

Reducing unnecessary hospital readmissions for patients with kidney failure requiring dialysis is an important priority for hospitals, nephrologists, and outpatient dialysis centers. Dialysis receiving patients have overall higher 30-day hospital readmission rates compared to others. The US Renal Data System 2020 Annual Data Report shows 30-day readmission rates for Medicare beneficiaries aged greater than or equal to 66 years were 16.6% for individuals without chronic kidney disease, 23.2% with chronic kidney disease, and 31.1% with patients receiving dialysis.^1^

Predictors of readmissions in patients receiving are similar to other diseases with some differences regarding dialysis-specific factors. Dialysis-related factors include dialysis vintage, vascular access type, missed dialysis treatments, dialysis treatment characteristics, failure to achieve target weight, intradialytic hypotension, and discharge to a nonhospital-affiliated dialysis center.^2^

Using readmission risk models to identify which patients receiving dialysis may be at higher risk for readmission can help clinical teams focus resources efficiently on the transitions of care for those patients to reduce unnecessary readmissions.^2^ In this retrospective analysis, we examined the performance of the Epic readmission risk model (version 1) performance for adult patients receiving dialysis discharged from Duke University Health System (DUHS).

We performed a retrospective study of the performance of the Epic unplanned readmission risk model for adult patients with kidney failure receiving dialysis discharged from DUHS hospitals from May 1, 2017 to April 30, 2021. This readmission risk model has been investigated in our general medicine patient population previously and reported separately.^3^ Further risk model details, risk thresholds, patient exclusionary criteria, definitions of renal disease with dialysis, and definitions of readmissions are detailed in Item S1.

Table 1 shows that, consistent with known demographics and compared to other general medicine patients, our patients receiving dialysis have a higher percentage of Black race, male sex, public insurance (eg, Medicaid), higher length of stay, and higher readmission rates and risk scores. Table 2 shows the area under the receiver operating characteristic curve for the readmission risk score was worse (*P* < 0.004) for patients receiving dialysis (0.681) versus general patients (0.705). However, the high-risk threshold has very good sensitivity (71%) for patients receiving dialysis, while still maintaining a relatively high positive predictive value (33%). When examining model performance across age, sex, race, and ethnicity, aside from slightly worse performance among older individuals, model performance was comparable (Table S1). Also, the area under the receiver operating characteristic curve for the model during the pre-COVID era was 0.684 (0.666-0.702) and the area under the receiver operating characteristic curve for the COVID era was: 0.676 (0.648-0.704), suggesting no meaningful difference of the pandemic on model performance.

**Table 1:** Baseline Characteristics

	Patients Not Receiving Dialysis	Patients Receiving Dialysis
Number (%)	162,308 (96%)	6,714 (4%)
Age, y (median, IQR)	63 (50-73)	61 (51-70)
Male sex, *n* (%)	78,747 (48%)	3,664 (55%)
Race, *n* (%)		
Non-Hispanic white	97,151 (60%)	1,549 (23%)
Non-Hispanic black	49,972 (31%)	4,618 (69%)
Hispanic	5,522 (3%)	243 (4%)
Other/unknown	9,663 (6%)	304 (5%)
Insurance, *n* (%)		
Private	43,885 (27%)	541 (8%)
Public	110,276 (68%)	6,114 (91%)
Other/unknown	8,147 (5%)	59 (1%)
Inpatient clinical service, *n* (%)		
General medicine	67,359 (42%)	4,539 (68%)
Heart	22,051 (14%)	728 (11%)
Oncology	7,802 (5%)	69 (1%)
Orthopedics	17,597 (11%)	60 (1%)
Surgery	21,971 (14%)	720 (11%)
Transplant	2,107 (1%)	216 (3%)
Other	23,241 (14%)	382 (5%)
Length of Stay, d, median (IQR)	4.0 (2.3-7.0)	5.1 (3.1-9.0)
Maximum risk score, median (IQR)	13 (9-19)	26 (19-37)
Readmission rate (overall)	11%	25%

Abbreviation: IQR, interquartile range.

**Table 2:** Epic Readmission Risk Model Predictive Performance

	Patients Not Receiving Dialysis	Patients Receiving Dialysis
Positive predictive value (high-risk group)	27% (0.262-0.273)	33% (0.317-0.346)
Sensitivity (high-risk group)	32% (0.318-0.332)	71% (0.687-0.732)
Specificity (high-risk group)	88% (0.883-0.886)	52% (0.504-0.531)
AUROC	0.705 (0.701-0.709)	0.681 (0.667-0.697)

*Note:* Measures of uncertainty in table are 95% CI.

Abbreviation: AUROC, area under the receiver operating characteristic curve.

In this report, we show that while the Epic readmission risk model has worse performance in patients receiving dialysis, because of the overall higher readmission rate in the dialysis population, the score is more clinically useful. Our high-risk grouping has greater sensitivity (71% vs 32%) and positive predictive value (33% vs 27%) among patients receiving dialysis. This allows patients receiving dialysis to receive more interventions with a higher degree of accuracy. These interventions could include nonspecific processes that affect readmission reduction for all high-risk patients as well as those specific to patients receiving dialysis. At DUHS, nonspecific interventions for all general medicine patients at high risk for readmissions include arranging postdischarge community resources, follow-up appointments within 7 days of discharge, pharmacy support in discharge medication reconciliation, and postdischarge follow-up phone calls by our Resource Center after discharge. For patients receiving dialysis, the literature supports other interventions such as more frequent nephrologist visits, anemia and electrolyte management, nutritional assessment, intensive medication management, dry weight achievement, better blood pressure control, and case management support.^4^, ^5^, ^6^

Although our internal analysis suggests that the Epic readmission model is useful for patients receiving dialysis, there is room to improve performance. However, although one can develop population-specific scores with better performance, this often comes at a higher implementation and maintenance cost. This work also highlights the value of not just considering global performance metrics like the area under the receiver operating characteristic curve, but also decision rule performance like sensitivity and positive predictive value.

Limitations of this work include being unable to account for readmissions occurring at other facilities outside of DUHS. In order to track readmissions at non-DUHS hospitals, we would need claims-based data which we did not have access to. As to this impact, assuming that patients readmitted at non-DUHS hospitals would have experienced less care coordination, it is not unreasonable to attribute a higher readmission rate to that subpopulation, which means our data is most likely underestimating readmission rates and positive predictive values for this model. Another limitation is the risk model is “off the shelf” and not customized to our institution. Given regional and state differences in readmissions and model performance and lack of other institutions’ publishing their experience with the Epic model, these findings may not be generalizable to other hospital or health systems and those entities should consider analyzing the performance of the Epic model if desiring to apply it in readmissions reduction work.

## Supplementary Material

Supplementary File (PDF) Item S1, Table S1.
