# Supplementary material for: A Readmission Risk Model for Hospitalized Patients Receiving Dialysis: Evaluation of Predictive Performance
Source: Kidney Med. 2022 Jun 24;4(8):100507. doi: 10.1016/j.xkme.2022.100507 (PMC9437601; doi:10.1016/j.xkme.2022.100507)
Supplement: Supplementary File (PDF) — Item S1, Table S1. [file mmc1.pdf]

Item S1: Supplementary Methods

Setting

This retrospective study analyzed the performance of the Epic readmission risk model (version 1) for adult patients (>= 18 years) with kidney failure receiving dialysis discharged from Duke University Health System (DUHS) hospitals from 5/1/2017 to 4/30/2021. DUHS is located in North Carolina and consists of three hospitals: one tertiary (Duke University Hospital, DUH, Durham, NC, USA) and two community (Duke Regional Hospital, DRH, Durham, NC, USA and Duke Raleigh Hospital, DRAH, Raleigh, NC, USA). There are approximately 1,500 inpatient beds across all three hospitals. Since 2014, we have used a shared, Epic-based electronic medical record (EMR) system.

Electronic Medical Record and Risk Model

The Epic unplanned readmission risk model (version 1) is a component of the Epic EMR and has been running continuously for us at DUHS since November 2017. The Epic readmission risk model was derived from data from 4 hospitals and used LASSO penalized regression techniques to produce the final model[1]. Final model variables include patient age, clinical diagnoses variables, laboratory variables, medication numbers and classes, order types, and utilization variables. Renal failure as a variable is captured within the model by laboratory values (creatinine) and the diagnosis of renal failure. The EPIC unplanned readmission risk model has been investigated on our general medicine patient population previously and reported out separately[2]. This risk model calculates a score every 4 hours for readmission risk for inpatients. The score is a continuous variable from 0 to 100 which increases with readmission risk but the

score does not assign a specific probability. The score is readily available to clinical inpatient teams on their patient lists.

We used the maximum risk score for each patient hospitalization and set the high-risk threshold to identify the top quartile at risk for readmission for all patients, regardless of diagnosis. Based on case management and clinical provider feedback, we estimated that supplementary interventions for preventing readmissions could be accomplished in 25% of all discharges. Therefore, we set the score threshold for high risk based on capacity to provide the intervention, rather than metrics of test performance.

Variables

We measured and reported raw unplanned 30-day readmission rates from 5/1/2017 – 4/30/2021. Data on index hospitalizations and hospital readmissions was extracted from Epic EMR database. 30-day readmissions include patients discharged from a DUHS hospital after an inpatient hospitalization and are readmitted to a DUHS hospital within 30 days of the index hospitalization discharge. Planned readmissions were identified using DUHS billing systems which interpreted readmissions as planned or unplanned, mirroring the CMS algorithm[3]. We excluded patients whose index admissions were due to psychiatric diagnoses, sickle cell disease, rehabilitation care, non-surgical cancer diagnoses, were admitted for inpatient hospice, were transferred to other acute facilities, died during the index hospitalization, had left against medical advice, or had a planned readmission. Patients with sickle cell disease have a unique care plan system at DUHS that involves separate risk assessment and interventions, hence the logic for excluding them in the model analysis. The rest of the exclusions are consistent with the CMS algorithms for defining readmissions[3].

We categorized patients as chronic kidney disease on dialysis based on ICD-10 code (N18.6) and a CPT code for administration of hemodialysis (90935 or 90937) at the index encounter, along with ICD-10 code or problem list code of N18.6 at a previous encounter. An internal chart review (performed by DG) of 10 charts showed perfect positive predictive value (PPV) with this definition. For patients with renal disease requiring hemodialysis, the baseline characteristic variables analyzed included age, race, ethnicity, insurance type, inpatient discharging clinical service, length of stay, readmission rates, and Epic readmission risk maximum scores.

Statistical Analysis

We summarized patient demographics stratified on kidney failure requiring dialysis as percentages and medians for categorical and continuous variables respectively. We evaluated the performance of the readmission risk score based on the overall area under the receiver operator characteristic (AUROC), sensitivity, specificity, and PPV of our high-risk threshold. Statistical software used in the analysis was R software version 4.0.1.

Ethics and Institutional Review

This study was determined by the Institutional Review Board (IRB) of Duke University as exempt without the need for informed consent because it constitutes a review of de-identified records.

Table S1: Model Performance by Baseline Demographics

	AUC	95% CI
Male	0.675	(0.655, 0.696)
Female	0.689	(0.667, 0.710)
>= 65	0.640	(0.614, 0.665)
< 65	0.701	(0.683, 0.720)
Non-Hispanic White	0.640	(0.607, 0.672)
Non-Hispanic Black	0.692	(0.675, 0.710)
Hispanic	0.696	(0.619, 0.774)

References

1. Epic. *Cognitive Computing Model Brief: Risk of Unplanned Readmission (Version 1).* 2016, pp. 1–13. Available online: www.Epic.com (accessed on 12 June 2020).

2. Gallagher D, Zhao C, Brucker A, Massengill J, Kramer P, Poon EG, et al. *Implementation and Continuous Monitoring of an Electronic Health Record Embedded Readmissions Clinical Decision Support Tool.* J Pers Med. Aug 26 2020;10(3) doi:10.3390/jpm10030103

3. Center for Medicare and Medicaid Services (CMS). Hospital Readmissions Reduction Program (HRRP). Updated 2020, February 11. Accessed 06/16/2020, https://www.cms.gov/Medicare/Medicare-Fee-for-Service-Payment/AcuteInpatientPPS/Readmissions-Reduction-Program
